# Postural correlates of visual incentives: application to food and alcohol stimuli

**DOI:** 10.3389/fpsyg.2025.1612425

**Published:** 2025-11-03

**Authors:** Duygu Duman, Sumeyye Kızılışık, Amel Zitouni, Salvatore Campanella, Thierry Lelard, Mbarka Akounach, Harold Mouras

**Affiliations:** ^1^UR-UPJV4559, Laboratoire de Neurosciences Fonctionnelles et Pathologies, UFR de Médecine, Université de Picardie Jules Verne, Amiens Cedex, France; ^2^Laboratoire de Psychologie Medicale et d’Addictologie, ULB Neuroscience Institute (UNI), CHU Brugmann-Universite Libre de Bruxelles (U.L.B.), Bruxelles, Belgium; ^3^UR-UPJV3300, Adaptations Physiologiques à l’Exercice et Réadaptation à l’Effort, UFR des Sciences du Sport, Université de Picardie Jules Verne, Amiens, France

**Keywords:** posturography, embodiment, incarnation, incentives, motivation, food, alcohol

## Abstract

**Introduction:**

Alcohol consumption is a major public health concern, linked to numerous diseases, including neurological disorders. Building on research into the interaction between emotion and motor processes, posturography has proven useful for studying how socioaffective information influences body sway. Few studies have explored this under motivational conditions, with limited work on food or alcohol cues.

**Methods:**

Fifty-five healthy participants viewed visual stimuli in four conditions (neutral–alcohol, alcohol, neutral–food, food) while their postural responses were recorded. After the experiment, participants rated each stimulus on pleasantness, unpleasantness, consumption, approach, avoidance, and intensity.

**Results:**

Subjective ratings differed significantly between conditions. Postural variability and movement amplitude were modulated by both incentive type (food vs. alcohol) and valence (incentive vs. neutral). Notably, food cues increased postural movement, whereas alcohol cues decreased it.

**Discussion:**

These findings highlight distinct motor signatures for different appetitive cues and contribute to understanding how emotions and motivation shape embodied responses to environmental stimuli.

## 1 Introduction

From the very beginnings of ethology, the category of “motivated behaviors” has been recognized as including those designed to ensure the survival of the species (Eibl-Eibesfeldt and Kramer, 1958). Since, by definition, these behaviors somehow require interaction with the environment to reach a target in order to ensure survival (and incidentally obtain a reward), they implicitly articulate a strong interaction (notably at the cerebral level) between emotion and motricity (Mogenson et al., 1980; Lang, 2014). Emotion can be thought of as a predisposition to action (Lang, 2014), inducing context-dependent behavior (e.g., approach-avoidance) mediated by responses that may be automatic (Campbell et al., 2013; Pankseep and Biven, 2012). According to this point of view, we would be inclined to move toward that which promotes our survival and well-being, and to move away from potentially traumatic experiences, including painful ones (Elliot and Thrash, 2001). This dichotomy was the basis for the construction of experimental banks of emotional images classified as “appetitive” or “defensive” based on the subjective approach-avoidance behavior induced (representing the modulation of the distance between the subject and the target; Lang et al., 1997). According to several motivational theories, approach behavior is triggered by appropriate incentives (Aagmo, 1999; Singer and Toates, 1987). According to Bindra (1974), this involves “a hypothetical set of neural processes that promote goal-directed actions about particular classes of incentive stimuli” defined as a *Central Motive State*. In particular, it is hypothesized that an incentive may guide a response selection process via the excitatory (even priming) influence of the motive on locomotor actions. Therefore, as Frijda (1986) posits, motivation will elicit a behavioral system (a sequence of potential actions) by appropriate external stimuli.

In the incentive category, food and alcohol are at the forefront. Regarding food a number of studies have investigated automatic approach tendencies (approach bias) in response to the presentation of food pictorial stimuli. Food approach-avoidance processes have been shown to be influenced by a number of factors with (i) the *state of satiety* altering the perception of peripersonal space (Bertonatti et al., 2021); (ii) *food sensitivity* (trait or state, i.e., craving) affecting approach-bias toward food (Brignell et al., 2009; Brockmeyer et al., 2015); (iii) the *perceived hedonistic value* of food (di Pellegrino et al., 2011); (iv) *learning* to avoid an automatic approach bias towards chocolate (Schumacher et al., 2016). On a clinical level, Frausto et al. (2022) showed in *anorexia nervosa* neuronal markers of early attraction (50–300 ms) followed by rapid suppression in response to food. In *Binge Eating Disorder* with overweight, there is a conflict between a positive conscious evaluation and automatic avoidance (ambivalence) towards food (Leehr et al., 2016; Paslakis et al., 2017). In the scientific literature, various tasks have been developed to quantify implicit attitudes in response to food stimuli (Czyzewska et al., 2011; Kakoschke et al., 2019). Regarding alcohol, approach-avoidance attitudes have been documented in different studies but mainly through the prism of addiction. Barkby et al. (2012) highlight the motivational conflict that exists in addicted patients (desire vs. willingness to abstain), but which does not seem to be reflected in the automatic processes measured. Cousijn et al. (2014) show an increased approach bias towards alcohol in a negative emotional context, linked to a difficulty in avoiding alcohol-related stimuli rather than an acceleration of the approach. Imaging methods (fMRI; Wiers et al., 2014) shows that in addicted patients, brain regions involved in motivation and reward are over-activated, while regions involved in cognitive regulation are weakened.

The exploration of approach-avoidance behaviors (seen as part of an overall motivational-behavioral response) has greatly benefited from the extension of observations to the neural and peripheral correlates of these behaviors. For food, Rossi et al. (2017) have shown that implicit activation of oral movements such as swallowing (vs. expectoration) increases positive (vs. negative) perception of taste in a variety of languages and media. These physiological activations, quasi-automatic and accompanying the processing of appetitive food stimuli, are an integral part of grounded-cognition approach, placing this embodiment, this physiological resonance, at the center of the behavioral response (Papies et al., 2020). For alcohol, this resonance may appear to be less important, since the social dimension of alcoholic stimuli appears to be more important than for food (Papies et al., 2020). This resonance/synchrony between approach-avoidance type behaviors and somatic/physiological variations (including the motor and postural responses) is at the foreground of the concept of “embodied motivation” (Harmon-Jones et al., 2013). In particular, the link between the approach bias towards incentives commonly evoked in motivational neuroscience and its motor correlates is central to this theoretical concept. Recently, Solzbacher (2022) showed in an approach-avoidance task faster reactions under congruent conditions (approaching positive stimuli, avoiding negative stimuli) only when using a joystick and not simple buttons, demonstrating the embodiment of the automatic approach-avoidance bias underlining the central role of action-associated movements for behavioral responses. Rougier et al. (2018) have shown the importance of whole-body perception (and not just arm perception, for example), i.e., self-perception of approach-avoidance behaviors. Recently in virtual reality, Lee et al. (2021) report on the effects of stimulus valence and arousal on object selection behaviors measured from physiological responses, notably ocular responses. A recent study (Kaczmarek et al., 2019) shows a certain variability in the valence association with, for example, an avoidance response associated with anger (contrary to other results) and wonder being an emotion where approach motivation dominates, more than positive valence, and thus demonstrates the complexity of the link between emotion and behavioral response beyond a simple positive emotion/approach and negative emotion/avoidance association. Price et al. (2012) have shown that body posture precociously modulates startle eye blink and neuronal reactions, and in particular automatic emotional responses. Krieglmeyer et al. (2010) have obtained results supporting the idea of an automatic link between emotional valence perception and approach-avoidance behavior independent of conscious intentions or evaluation. In recent years, posturography has made it possible to study the motor and neural correlates of socioaffective information processing in different functional contexts (Lelard et al., 2019). Curiously, its use in response to the presentation of motivational stimuli remains largely exploratory. Within the framework of sexual incentives, we have demonstrated (Mouras et al., 2015) using posturography a freezing-type behavior in response to sexually explicit stimuli. For food, to our knowledge, only one study recorded postural modulation in response to food stimuli (Brunyé et al., 2013). In this study, participants tended to lean toward (approach) the foods they preferred and away from those they disliked (movements along the anteroposterior axis increasing over time, not present in the mediolateral direction). For alcohol, Noël et al. (2021) showed differences in postural tone between patients who relapsed after leaving treatment (backward movements in relation to alcohol-related stimuli and forward movements when exposed to sexual stimuli) and those who remained abstinent. As a result, this field remains virtually untouched.

Given that (i) the field of investigation in which this study is situated is virtually virgin; (ii) a large body of research has shown the central importance of the embodiment (including motor processes) in the deployment of approach-avoidance behaviors; (iii) that posturography appears to be a technique of interest for capturing this embodiment in different functional contexts and that this embodiment has been discussed taking into account its temporality between an early component and a late component (which refers to the recent proposal to move from the classic dual processes model for motivation to an iterative process allowing the modulation of early responses), our objectives were to explore for the first time the respective differential modulation of postural control by two different types of motivational incentives, food and alcohol. A secondary objective was to see if this modulation was correlated with the level of alcohol consumption as measured by standardized questionnaires.

In this study, food and alcohol stimuli were presented under identical experimental conditions, allowing them to be treated as comparable motivational targets. This design choice, grounded in embodiment and approach–avoidance theories, ensured a controlled comparison between two categories of appetitive cues. It enabled us to examine, within the same paradigm, whether stimuli from distinct but comparable motivational domains would elicit similar or divergent postural responses, without assuming such differences *a priori*.

## 2 Methods

### 2.1 Participants

A total of 65 participants (34 women, 31 men; mean age = 24.98 ± 7.21 years) were included in this study. Participants were invited to take part in the research through flyers, newspaper advertisements and posters. To enhance motivation and encourage voluntary participation, each participant received a €50 gift voucher as compensation for their involvement in the study.

To be eligible for participation, individuals had to meet specific inclusion criteria. Participants were required to be healthy, right-handed, have no diagnosed psychiatric disorders, not be using psychiatric medication, and have abstained from alcohol consumption in the 24 h preceding the experiment. Those who met these criteria were invited to the laboratory and signed a written informed consent form before participating in the study.

**Figure 1:**
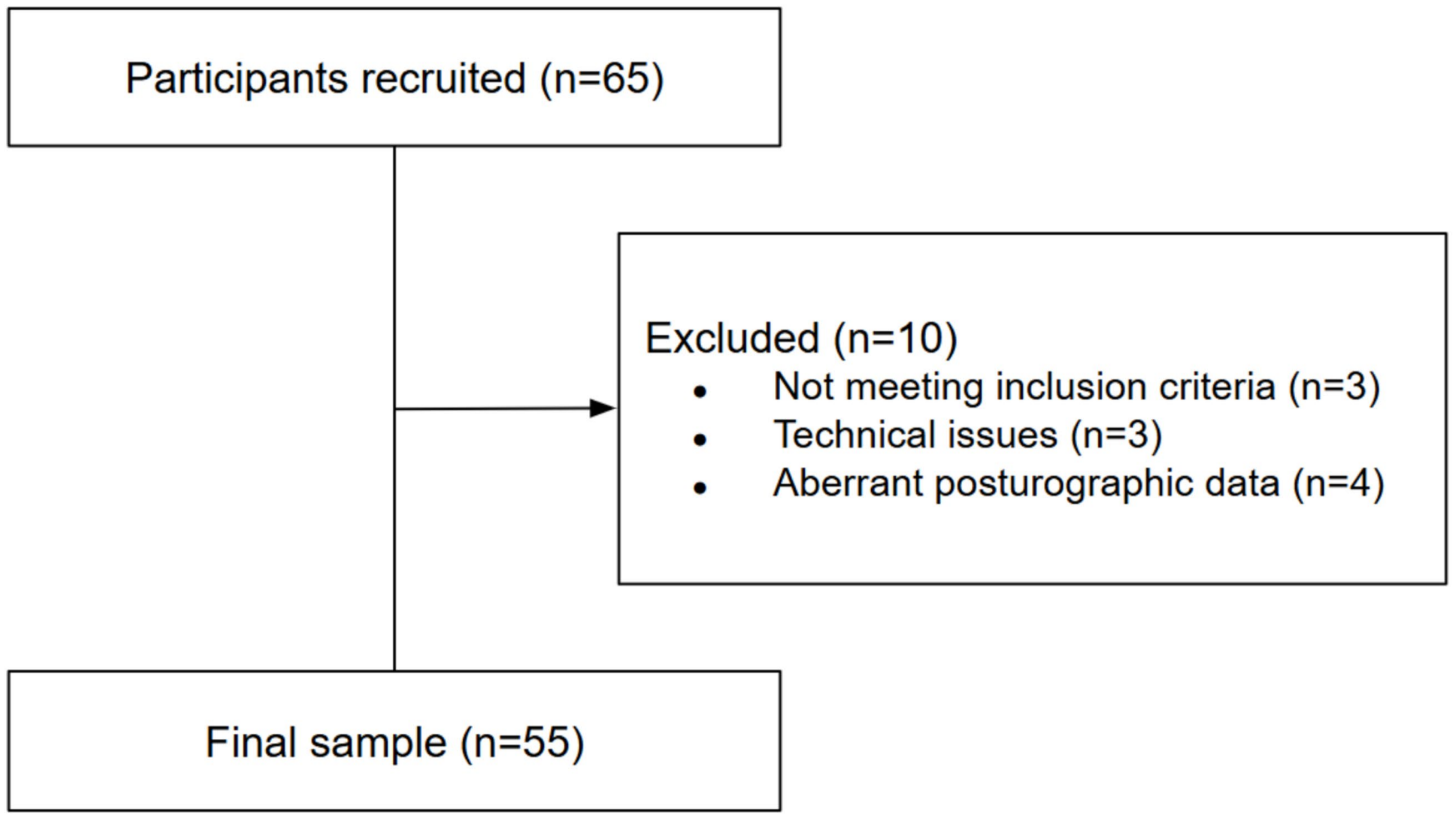
Flowchart of participant recruitment and exclusions.

Three participants were excluded from the study on predefined exclusion criteria: one due to alcohol consumption within the 24-h pre-experiment period, one due to left-handedness and another due to antidepressant medication treatment. This adjustment was made to ensure data reliability and maintain compliance with the study criteria. Three others due to technical incidents during the experiment, and a further four during data pre-processing, due to extreme postuographic data. As a result, data from 55 participants (28 men; mean age = 25 ± 5.75 years and 27 women; mean age = 25.19 ± 9.05 years) were retained for the final analysis. The recruitment and exclusion process of participants is summarized in Figure 1. A written informed consent form was signed by each participant.

The study protocol was approved by the Comité d’Ethique pour les Recherches Non Interventionnelles (CERNI, Université de Picardie Jules Verne, Amiens, France) and conducted in accordance with the Déclaration of Helsinki.

### 2.2 Stimuli selection

Visual stimuli were selected from three validated databases: the Cross-Cultural Food Image Database (CROCUFID; Toet et al., 2019), the Amsterdam Beverage Picture Set (ABPS; Pronk et al., 2015), and the Australian Beverage Picture Set (Onie et al., 2020). Four stimulus categories were created: Alcohol, Neutral Alcohol, Appetitive Food, and Neutral Food, each comprising 52 images matched for resolution, background, and viewing angle. Selection criteria ensured consistent visual presentation across categories, including standardized lighting and neutral backgrounds. Detailed descriptions of the databases, image characteristics, and selection procedures are provided in the Supplementary material (Stimuli databases selection procedure).

### 2.3 Measures

#### 2.3.1 Psychometric data

Prior to the experimental procedure, participants completed a series of questionnaires designed to assess various psychological and behavioral characteristics. These questionnaires collected sociodemographic information, including dominant hand, physical activity level, and the presence of any diseases or disorders, while also incorporating validated measures to evaluate psychological and cognitive functions. In this study, the French versions of seven widely used and scientifically validated questionnaires were administered. Participants’ depression levels were assessed using the Beck Depression Inventory-II (BDI-II), a widely used 21-item self-report questionnaire developed by Beck et al. (1996) to measure depressive symptoms and their severity (0–13: No depression, 14–19: Mild depression, 20–28: Moderate depression, 29–63 Severe depression). The State–Trait Anxiety Inventory (STAI-TRAIT), a 20-item anxiety assessment scale developed by Spielberger et al. (1971), was used to measure participants’ anxiety levels. The participant is asked to score these items from 1 (almost never) to 4 (almost always). The State–Trait Anxiety Inventory (STAI) scores were used for descriptive purposes only and were not part of the formal exclusion criteria. A high score on the STAI reflects a self-reported level of anxiety at the time of testing (state) or as a general tendency (trait), but it is not, in itself, a clinical diagnosis. Participants with elevated anxiety scores were retained in the sample, as such scores can occur in healthy, non-clinical populations and may reflect transient emotional states or personality-related tendencies rather than pathological anxiety. The present study aimed to recruit a non-clinical population without diagnosed psychiatric disorders, rather than to exclude all individuals with above-average psychometric scores.

The Mini-Mental State Examination (MMSE), a widely used 30-item test designed to assess cognitive functions such as thinking, communication, comprehension, and memory, was administered to participants in an interactive manner. The test, developed by Kurlowicz and Wallace (1999), consists of several sections, including Orientation, Learning, Attention and Calculation, Recall, Language, and Constructive Praxis. Participants responded to the examiner’s questions in each section, and their cognitive health was evaluated based on their responses. Participants’ hand dominance was determined using the Edinburgh Handedness Inventory (Oldfield, 1971). It is a 10-item questionnaire designed to assess an individual’s dominant hand. For each item, participants indicate which hand they use while performing a specific activity. Another questionnaire, the Dutch Eating Behavior Questionnaire (DEBQ), developed by Strien et al. (1986), was administered to assess eating disorders. This 33-item questionnaire evaluates three different eating behaviors in adults: emotional eating, external eating, and restrained eating. The participant’s alcohol consumption level is a crucial aspect of our study. Therefore, the Alcohol Use Disorders Identification Test (AUDIT; Williams, 2014) was administered, a 10-item questionnaire designed to diagnose risky alcohol consumption or alcohol use disorder. Participants rated almost all questions, except for Questions 9 and 10, on a scale from 0 (never) to 4. For these two questions, they selected one of three options: 0, 2, or 4. To distinguish between levels of alcohol use, participants were divided into two groups—low and high alcohol consumers—based on a median split of their total AUDIT scores. Finally, the Fagerström Test (Heatherton et al., 1991) was used to assess tobacco/nicotine addiction. Participants responded to six questions, with scores categorized as follows: 0–2 (no addiction), 3–4 (low addiction), 5–6 (moderate addiction), 7–8 (strong addiction), and 9–10 (very strong addiction).

#### 2.3.2 Postural data

Postural data for each condition were collected using an AMTI force platform (AMTI BP600 × 400, Watertown, MA, USA) equipped with four dynamometers. The MP150 Biopac system (Biopac Inc., Goleta, CA, USA) was employed to ensure the synchronized acquisition of postural and physiological data at a sampling frequency of 1,000 Hz.

The recorded voltage values were converted into Newtons using the following equation. Different analog sensitivity values were derived from the default parameters of the AMTI force platform, enabling the calculation of six measurements. These measurements included force components in the mediolateral (Fx), anteroposterior (Fy), and vertical (Fz) directions, as well as moments around the mediolateral (Mx), anteroposterior (My), and vertical (Mz) axes.

Subsequently, posturographic data were processed using a second-order Butterworth low-pass filter at 5 Hz to remove unwanted frequencies while preserving signal integrity. To further optimize data processing and reduce file size, the data were resampled to 100 Hz, ensuring their suitability for further analysis.

For each trial and image type, several postural measures were computed to assess body sway and stability. The Center of Pressure (COP) position was determined along both the anteroposterior (AP) and mediolateral (ML) axes, providing insight into weight distribution and balance control. Additionally, the standard deviations of COP in these directions were calculated to quantify the variability of postural sway. To further evaluate postural stability, the COP path length, representing the total displacement of body sway over time, was computed. These measures collectively provide a comprehensive analysis of postural control and stability under different conditions.

#### 2.3.3 Rating data

Following the completion of posturographic data recording, participants proceeded to the subjective evaluation phase, where they assessed visual stimuli presented under predefined experimental conditions. This phase aimed to measure the psychological and emotional responses elicited by each image, facilitating the investigation of the relationship between posturographic data and subjective evaluations.

The experiment included 208 visual stimuli across four conditions (“neutral alcohol,” “alcohol,” “neutral food,” and “food”), presented using E-Prime 3 software. Each stimulus was displayed for 3 s, followed by a sequence of six standardized questions, assessing Pleasantness, Unpleasantness, Approach Desire, Consumption Desire, Avoidance Desire, and Intensity on a 9-point scale. Participants provided responses via a computer keyboard with no time constraints but were encouraged to answer promptly.

The evaluation phase followed the same eight-session structure as the experimental phase, with 26 images per session and three-minute rest intervals between blocks.

### 2.4 Experimental paradigm

**Figure 2:**
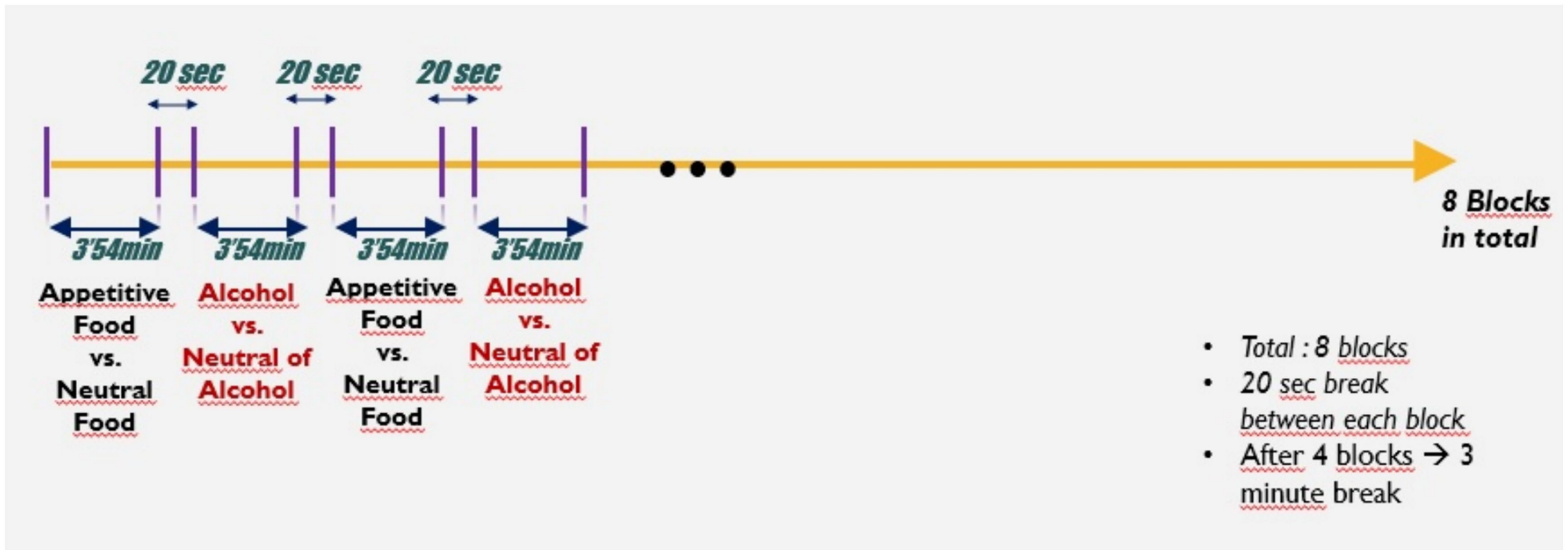
General visualization of the paradigm.

**Figure 3:**
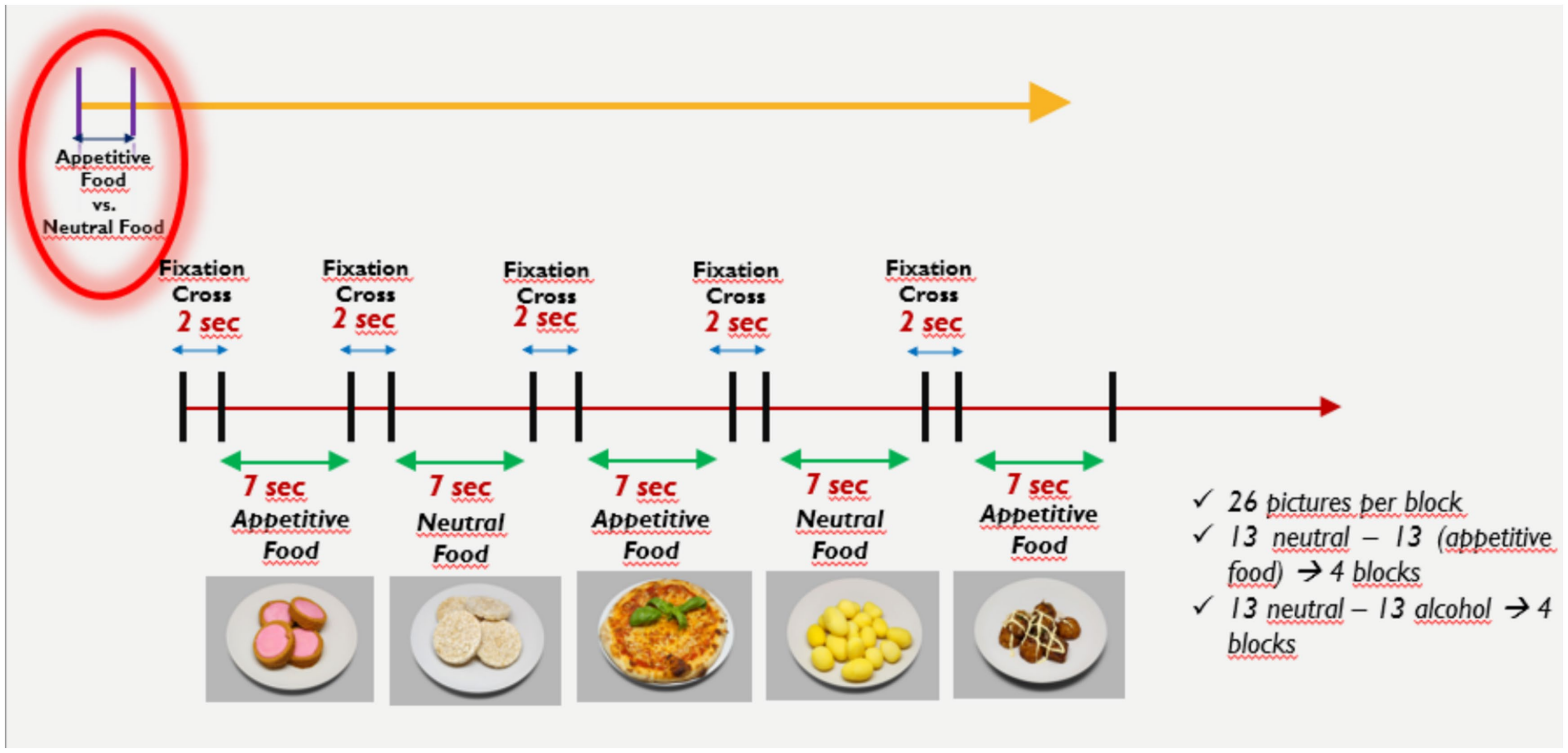
Visualization of 1 block of the paradigm.

The paradigm developed by Luther et al. (2023) was adapted for the present study. This research holds significant value as the first study to integrate EEG and posturography simultaneously. It investigated the effects of visual stimuli (pleasant/unpleasant) on individuals’ physiological responses (posturography) and brain activity (EEG). The primary objective was to examine how visuals with different valences influence electrical activity in the brain and to understand their impact on posture and balance. The final paradigm is illustrated in Figures 2, 3.

Although EEG signals were recorded simultaneously with posturographic data as part of a broader experimental protocol, the current manuscript focuses exclusively on postural and affective measures. The EEG data will be analyzed and presented in a separate publication addressing neural temporal dynamics in motivational processing.

Within the paradigm, 52 appetizing food, 52 neutral food, 52 alcohol and 52 neutral alcohol images were taken from the databases. There are a total of 208 images to be shown to the participant throughout the experiment. Unlike Luther’s paradigm, this paradigm was adopted as 7 s because the 2 s in which the stimuli are given may be insufficient to observe the changes that may occur in posturography in response to the stimuli. Longer durations are generally adopted in posturography studies (Akounach et al., 2022; Mouras et al., 2015). The experimental paradigm was created using E-prime. A visual of the paradigm created in Eprime is given in Figure 3. When the experimental paradigm is examined in general, the paradigm consists of 8 blocks. Each block contains 26 pictures. Thirteen of these pictures are neutral pictures and the remaining 13 are appetizing food or alcohol stimuli. The block with food visuals is followed by the block with alcohol stimuli. In other words, there are 4 blocks for food stimuli and 4 blocks for alcohol stimuli in the paradigm. There is a 20-s rest period between each block. This rest period is extended to 3 min between blocks 4 and 5. In the block content, there is a 7-s stimulus display followed by a 2-s fixation cross. The visuals in the block are randomly given. When all these periods are evaluated, the experimental paradigm lasts approximately 35 min.

Participants were taken to the room where the experiment would take place. They were then informed about the experiment and instructed to take the experimental posture on the posturography device. The E-Prime file designated for the experiment was executed, and simultaneously, the AcqKnowledge software was launched to record posturography data.

Following the approximately 35-min experimental session, participants were given time to rest. After the resting period, they were asked to rate the images they had seen during the experiment using a small keyboard, according to the criteria of Pleasant, Unpleasant, Consumption, Approach, Avoidance, and Intensity on a scale of 1 to 9. Once all sessions were completed, participants received gift cards and were asked to sign a document confirming their receipt of the gift cards.

### 2.5 Data processing and analyses

#### 2.5.1 Sample-size justification and power

Sample-size justification and power. We determined our target sample size based on an *a priori* power analysis conducted in GPower 3.1 for a repeated-measures ANOVA (within-subject factor with four levels: Alcohol, Neutral-Alcohol, Food, Neutral-Food). Assuming *α* = 0.05, desired power (1–*β*) = 0.80, a conservative non-sphericity correction *ε* = 0.75, and a small-to-medium effect size *f* = 0.20, the required sample size was N ≈ 52. Our analyzed sample (*N* = 55) meets and slightly exceeds this requirement. For completeness, we also report sensitivity for paired within-subject comparisons: with *N* = 55, α = 0.05 (two-tailed), and power = 0.80, the design is sensitive to within-participant effects of approximately dz. ≈ 0.38–0.40. All GPower inputs/outputs are provided in Supplementary material S2.

#### 2.5.2 At the group level

All statistical analyses were conducted using R software (R Core Team, 2021). For each of the six subjective evaluation dimensions (Pleasant, Unpleasant, Approach, Avoidance, Consumption, and Intensity), four experimental conditions were compared: Alcohol, Neutral-Alcohol, Appetizing Food, and Neutral-Food. In order to evaluate the suitability of the data for parametric analysis, Shapiro–Wilk normality test was applied for each condition and given rating value. Shapiro–Wilk test is a widely used and reliable method to test the normality of data distribution, especially in small and medium-sized samples. According to the obtained *p*-value, the results above 0.05 showed that the data was normally distributed, while those below 0.05 showed that it was not normally distributed. The groups whose sample numbers were outside this range were excluded from the analysis. Due to the non-normal distribution of the data, as assessed through visual inspection and confirmed by the Shapiro–Wilk normality test, the non-parametric Wilcoxon signed-rank test was used to compare the paired conditions. To control the risk of Type I errors arising from multiple comparisons, *p*-values were adjusted using the False Discovery Rate (FDR) correction method. A significance threshold of *q* < 0.05 was applied. Data for each dimension were visualized using boxplots, with statistically significant differences annotated using asterisks directly on the plots.

#### 2.5.3 Allowing to the level of alcohol consumption: at the subgroup level

This study focused exclusively on posturographic responses for global analysis. To assess participants’ levels of alcohol consumption, the Alcohol Use Disorders Identification Test (AUDIT) was administered. Based on the distribution of AUDIT scores within the sample, the median value was identified as 5. Using this threshold, participants were categorized into two groups: those with low levels of alcohol consumption (low group) and those with high levels of alcohol consumption (high group). In total, 30 participants were classified in the low group and 25 in the high group. Analyses comparing these two groups were conducted and are presented in the Supplementary materials section to maintain the coherence of the main text. These additional analyses provide valuable insights into the potential effects of alcohol consumption levels on posturographic responses, contributing to a more nuanced understanding of how individual differences in alcohol use may influence bodily stability and motor control under motivational stimulation.

## 3 Results

### 3.1 Psychometric data

In this study, psychometric data from a total of 55 healthy participants were analyzed. Sociodemographic characteristics and mean scores on the psychometric scales are summarized in Table 1. All participants reported predominantly using their right hand. Overall, the majority of participants exhibited low or non-clinical levels of depressive (BDI-II) and anxiety (STAI-T) symptoms, with no indication of clinically significant disordered eating behaviors (DEBQ).

**Table 1:** Means and standard deviations (in parentheses) for all 55 participants on the AUDIT, BDI-II, STAI-T, DEBQ, and Fagerström Test.

	Values (*n* = 55)
Sex (♂:♀)	28: 27
Age (years)	
Men	25 (5.75)
Women	25.19 (9.05)
AUDIT	6.25
BDI-II	8.89
STAI TRAIT	39.40
DEBQ restrained eating	2.12
DEBQ emotional eating	2.13
DEBQ external eating	3.13
Fagerström test	0.67

AUDIT: Alcohol Use Disorders Identification Test; BDI: Beck Depression Inventory-II; STAI: Trait Anxiety Inventory; DEBQ: Dutch Eating Behavior Questionnaire.

Alcohol use risk was evaluated using the Alcohol Use Disorders Identification Test (AUDIT). Based on the median score of 5, participants were divided into two groups: 30 participants were classified as low alcohol users, and 25 as high alcohol users. Overall, the sample consisted primarily of individuals with low or non-clinical levels of depressive symptoms and anxiety, with a small number of participants presenting moderate depression scores (*n* = 4) or high anxiety scores (*n* = 2). These cases were retained in the sample in accordance with our predefined inclusion criteria (see Methods).

Although the study primarily focuses on global analyses, the analyses based on AUDIT scores are presented in the Supplementary materials.

**Figure 4:**
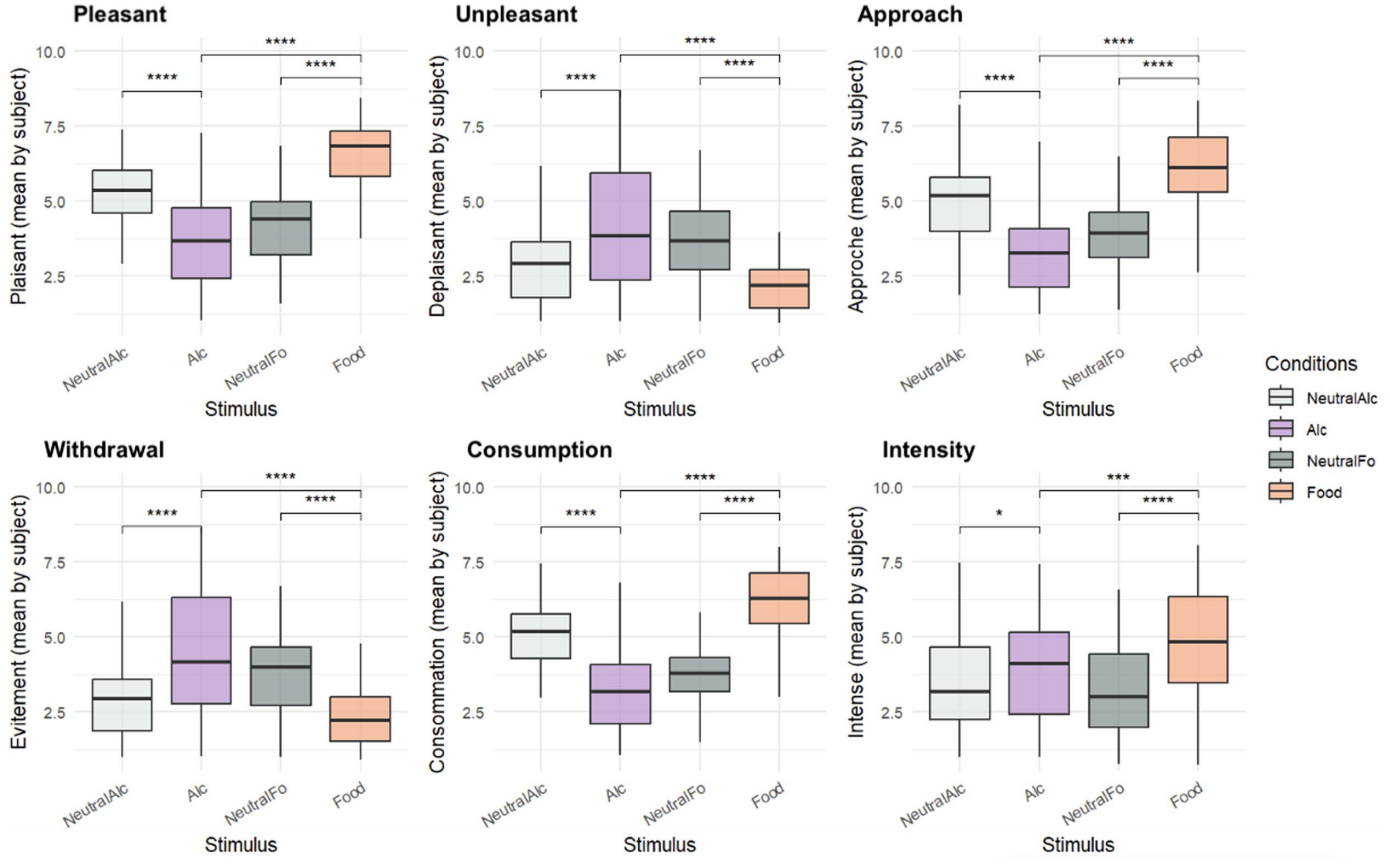
Subjective ratings by stimulus type: results from Wilcoxon Signed-Rank Test Mean (±SEM) subjective ratings across four stimulus categories for six motivational and affective dimensions. Ratings were given on a 1-to-9 Likert scale (Statistical significance: *p < 0.05, ***p < 0.001, ****p < 0.0001).

### 3.2 Subjective ratings

Statistical analysis using the Wilcoxon signed-rank test revealed consistent and significant differences across all affective dimensions. Figure 4 presents the subjective rating results. In the *Consumption* dimension, a significant difference was observed between Neutral-Food and Appetitive-Food (*W* = 0.0, *p* < 0.001, *p* = 7.06 × 10^−10^). In the *Pleasantness* dimension, a significant difference was observed between Neutral-Food and Appetitive-Food (*W* = 1.0, *p* < 0.001, *p* = 7.06 × 10^−10^). Similarly, in the *Approach Desire* dimension, Neutral-Food and Appetitive-Food differed significantly (*W* = 1.0, *p* < 0.001, *p* = 7.06 × 10^−10^). Significant differences were observed for all reported comparisons (all *p* < 0.001, see Table 2), with higher ratings for appetitive than neutral conditions.

**Table 2:** Subjective ratings across dimensions and stimulus conditions.

**Comparison**	**Dimension**	**Statistic**	***p*-value**	**Direction**
Neutral food vs. Appetitive food	Intensity	*W* = 63.0	*p* < 0.001	Appetitive > Neutral
Neutral food vs. Appetitive food	Unpleasantness	*W* = 0	*p* < 0.001	Neutral > Appetitive
Neutral alcohol vs. Alcohol	Consumption	*W* = 1405.0	*p* = 2.12×10⁻⁷	Alcohol > Neutral
Neutral alcohol vs. Alcohol	Avoidance	*W* = 206.5	*p* = 3.18×10⁻⁶	Neutral > Alcohol
Neutral alcohol vs. Alcohol	Approach	*W* = 1362.0	*p* = 1.08×10⁻⁶	Alcohol > Neutral
Neutral alcohol vs. Alcohol	Pleasantness	*W* = 1246.5	*p* = 3.18×10⁻⁶	Alcohol > Neutral
Neutral alcohol vs. Alcohol	Unpleasantness	*W* = 260.5	*p* = 6.25×10⁻⁵	Neutral > Alcohol
Neutral alcohol vs. Alcohol	Intensity	*W* = 476.5	*p* = 0.022	Alcohol > Neutral
Alcohol vs. Neutral food	ML Displacement	*F*-test	*p* = 0.00023	Alcohol > Neutral
Alcohol vs. Food	ML Displacement	*F*-test	*p* < 0.001	Food > Alcohol
Food vs. Neutral food	ML Displacement	*F*-test	*p* < 0.001	Food > Neutral
Neutral Alcohol vs. Neutral Food	ML Displacement	*F*-test	*p* < 0.001	NeutralAlc > NeutralFo

W = Wilcoxon signed-rank tests; F = repeated-measures ANOVA; ML = mediolateral displacement. Significant at *p* < 0.05; highly significant at *p* < 0.001.

These findings confirm the effective categorization of visual stimuli across both food- and alcohol-related conditions and demonstrate their differential affective impacts across a range of emotional dimensions.

### 3.3 Postural responses

**Figure 5:**
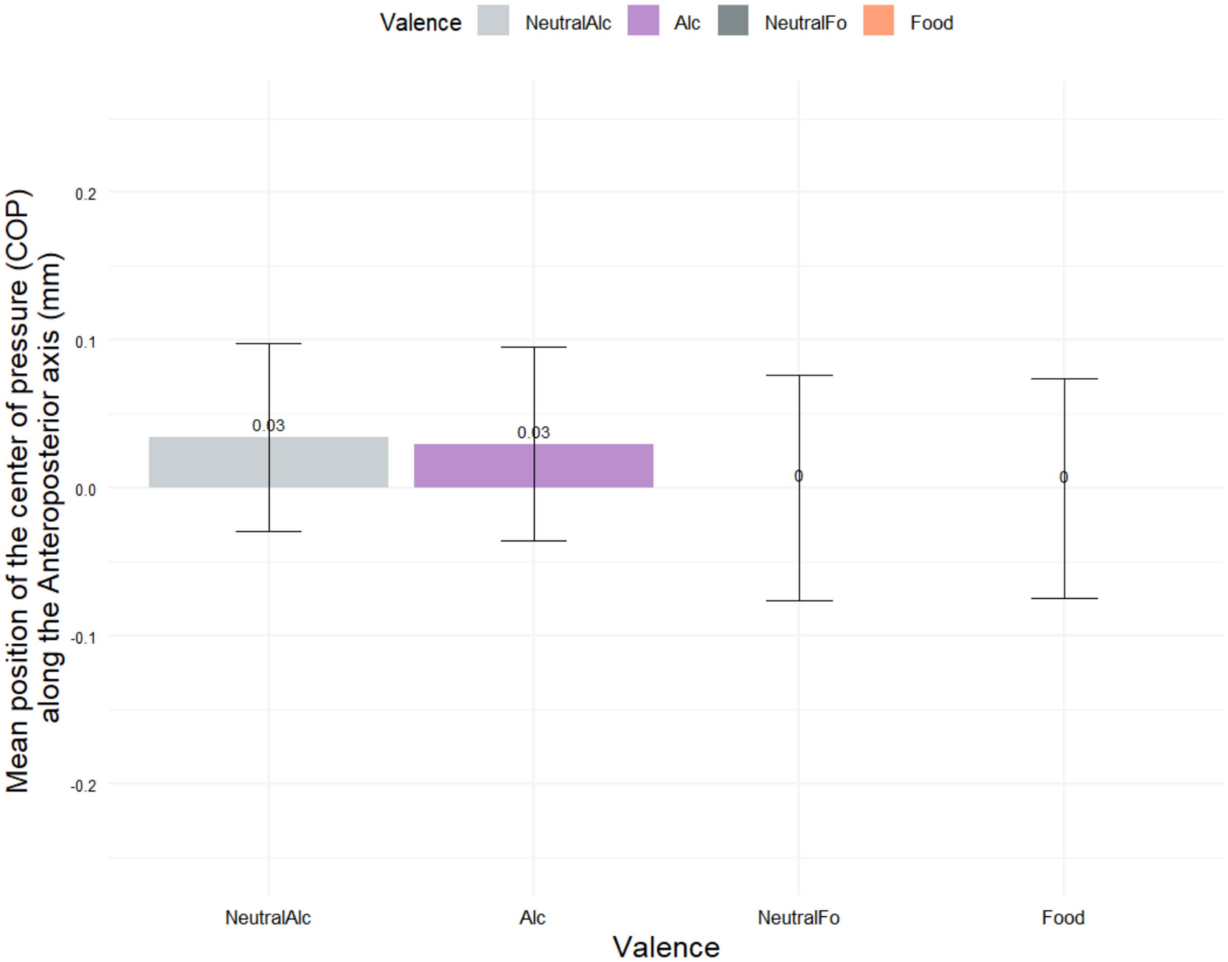
Mean center of pressure (COP) position along the anteroposterior axis (in mm) across four stimulus conditions (***p < 0.001).

Figure 5 presents the mean Center of Pressure (COP) positions along the anteroposterior (AP) axis for each of the four stimulus conditions: Alcohol (Alc), Neutral-Alcohol (NeutralAlc), Food, and Neutral-Food (NeutralFo). Descriptive analysis revealed a slight anterior shift for alcohol-related stimuli, with both Alcohol and Neutral-Alcohol conditions showing a mean value of 0.03 mm (±1.79 and ±1.73, respectively). In contrast, food-related conditions (Food and Neutral- Food) showed a mean COP value of 0.00 mm (±2.03 and ±2.08, respectively). However, statistical analysis indicated no significant differences between conditions (*p* = 0.9569), suggesting that the type of stimulus did not elicit distinct postural shifts along the anteroposterior axis.

**Figure 6:**
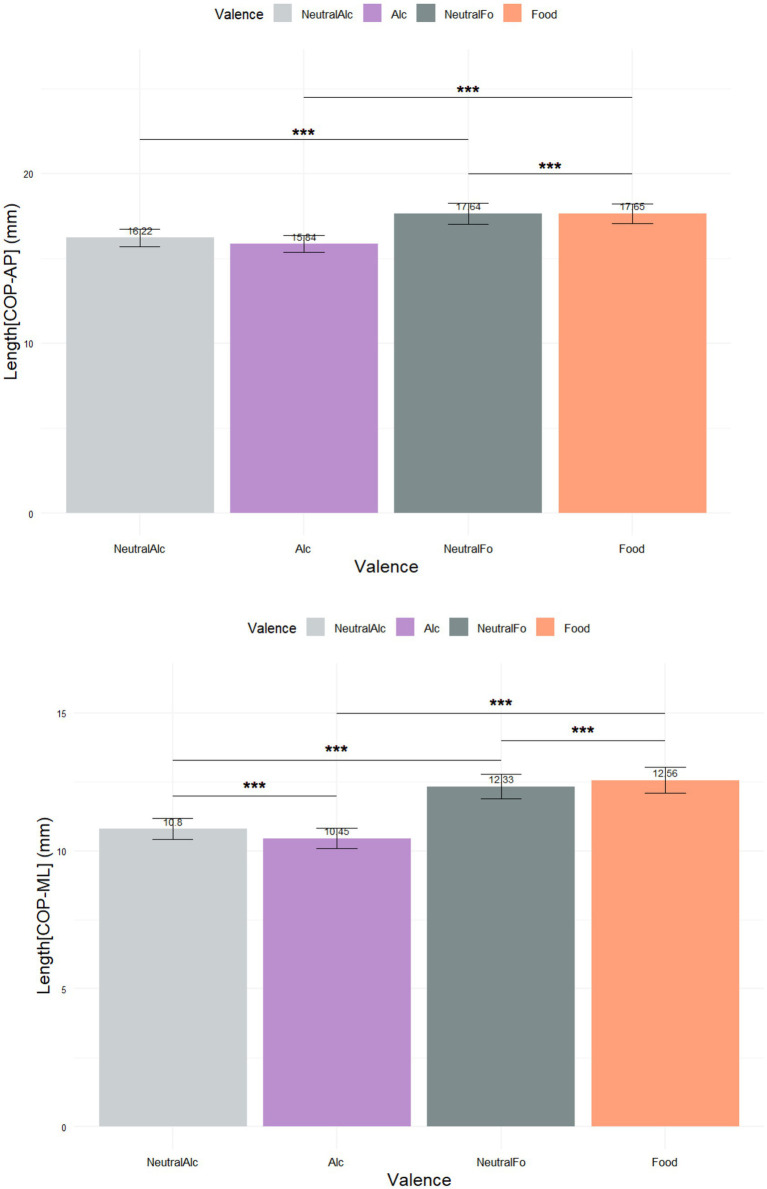
Mean center of pressure (COP) path lengths along the anteroposterior and mediolateral axes (***p < 0.001).

Figure 6 displays the mean Center of Pressure (COP) path lengths along the anteroposterior (2.a) and mediolateral (2.b) axes across four stimulus conditions.

The analysis of postural responses revealed significant differences in COP displacements across stimulus conditions. In the anteroposterior (AP) direction, a significant main effect of condition was found (*p* = 3.07 × 10^−5^). *Post hoc* Nemenyi tests showed that Alcohol stimuli led to significantly shorter COP-AP displacements compared to Food and Neutral-Food stimuli, while no significant difference was observed between Alcohol and Neutral-Alcohol. Food stimuli elicited significantly greater displacements than both Neutral-Alcohol and Neutral-Food conditions, and a significant difference was also found between Neutral-Alcohol and Neutral-Food, and a significant difference was also found between Neutral-Alcohol and Neutral Food.

Significant differences were observed for all reported comparisons (all p < 0.001, see Table 2), with higher ratings for appetitive than neutral conditions.

In contrast, no significant difference was found between conditions in terms of subjective valence ratings (e.g., Food: 0 ± 2.03; NeutralFo: 0 ± 2.08; Alc: 0.03 ± 1.79; NeutralAlc: 0.03 ± 1.73; *p* = 0.9569).

**Figure 7:**
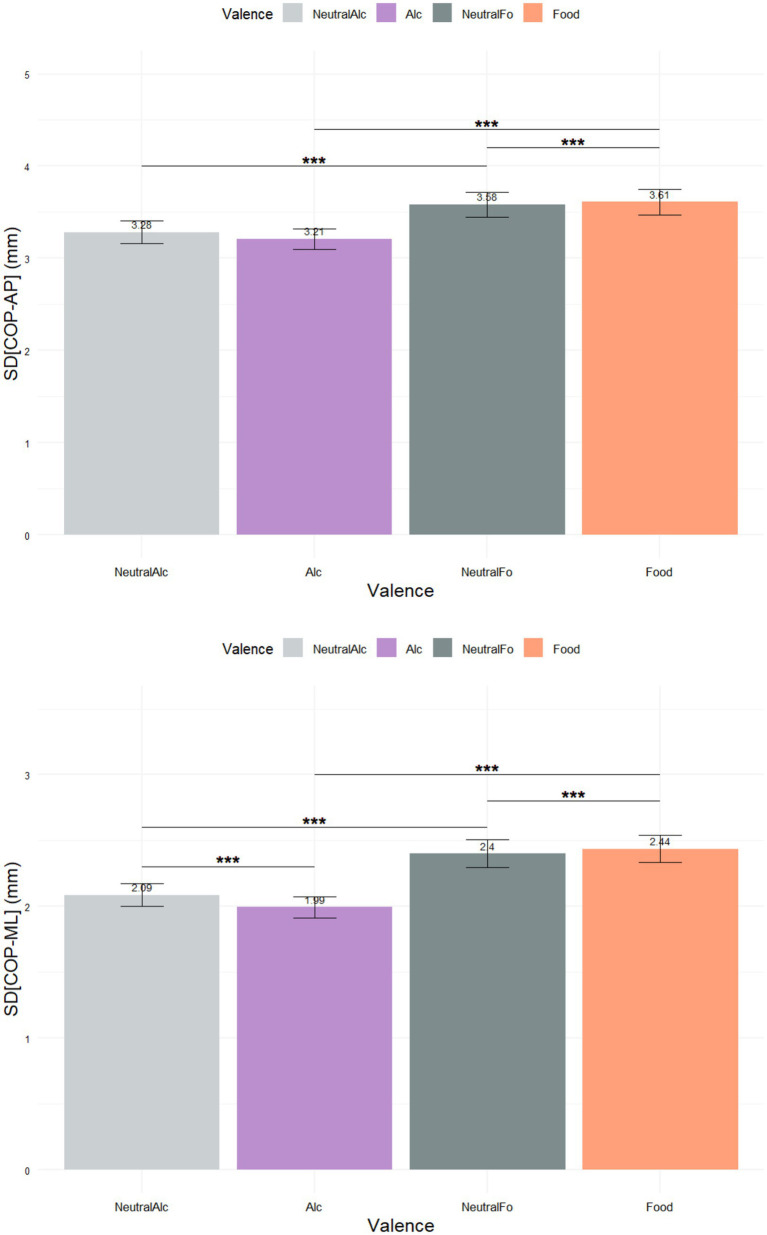
Standard deviation of center of pressure (COP) path lengths along the anteroposterior **(A)** and mediolateral **(B)** axes (***p < 0.001).

The standard deviation values of postural sway varied significantly according to the type of stimulus presented (Figure 7). In both the anteroposterior (SD(COP-AP)) and mediolateral (SD(COP-ML)) axes, Food and Neutral-Food stimuli were characterized by higher postural sway compared to Alcohol and Neutral-Alcohol stimuli. SD(COP-AP) values increased particularly in response to food-related stimuli, suggesting a decrease in anterior–posterior stability. Similarly, the medio-lateral path length showed significant variation across stimulus types. Post hoc Nemenyi tests revealed statistically significant differences between all conditions, indicating that each stimulus type elicited distinct postural responses. The mean SD(COP-ML) values were 2.43 ± 2.80 mm for the Food stimulus, 2.40 ± 2.81 mm for Neutral-Food, 2.00 ± 2.19 mm for Alcohol, and 2.08 ± 2.35 mm for Neutral-Alcohol. These findings suggest that food-related visual stimuli induce greater postural instability compared to alcohol-related stimuli, highlighting the sensitivity of postural control to the sensory and motivational content of the stimuli.

**Figure 8:**
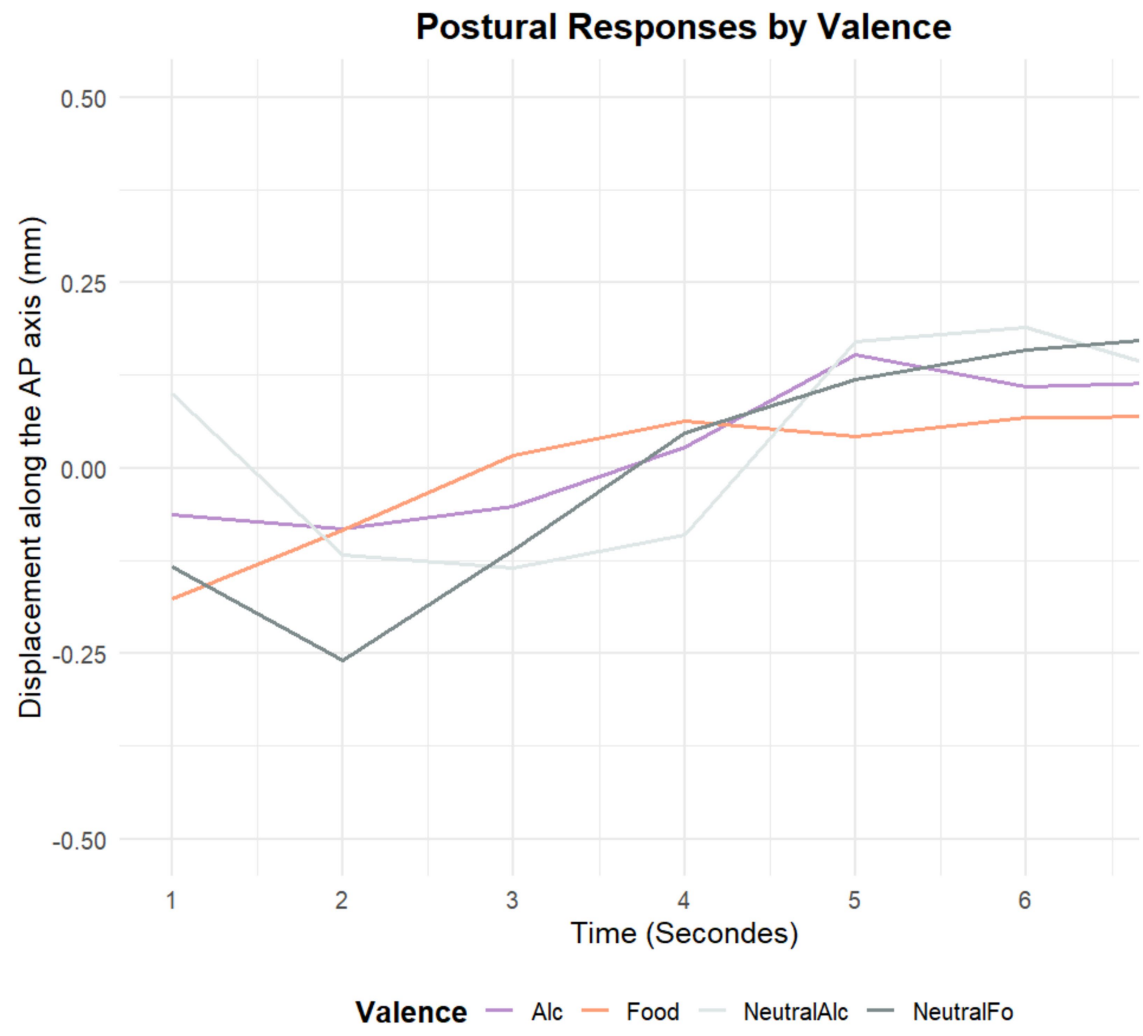
Time course of postural displacement along the antero-posterior (AP) axis in response to stimuli of different valence categories.

Figure 8 presents the temporal changes in postural displacement along the antero-posterior (AP) axis in response to stimuli with different valence categories: alcohol-related, food-related, and their neutral counterparts. The results reveal a general trend of increasing forward displacement across all conditions over the 7-s period. However, this effect is more pronounced for motivationally salient stimuli, particularly in the Alcohol and Food conditions, which show a steeper increase in positive AP displacement after approximately 4 s. In contrast, neutral conditions —especially Neutral-Food— initially show a backward shift before gradually moving forward. These findings suggest that alcohol- and food-related cues elicit stronger approach-related postural responses compared to neutral stimuli, indicating enhanced motivational and attentional engagement with these categories.

## 4 Discussion

As a large number of experimental and theoretical scientific studies have emphasized the importance of incarnation/embodiment in the development of behavioral responses to motivational targets (particularly involving approach-avoidance behavior), the use of posturography to measure behavioral biomarkers in response to motivational targets seems particularly appropriate. However, very few studies have used posturography to examine the physiological correlates in response to the presentation of motivational images evoking food and alcohol. This study is therefore the first to have used these two types of incentives together in a single experiment.

### Subjective ratings

Regarding subjective data, they were in accordance with our hypothesis for food: in response to food as compared to neutral-food stimuli, we report significantly higher scores in the Pleasant, Approach, Consumption and Intense dimensions. On the contrary, in response to alcohol as compared to neutral-alcohol stimuli, significant lower ratings were recorded for the Pleasant, Approach and Consumption dimensions. This means that neutral-alcohol stimuli (despite being chosen as neutral) had higher mean values compared to alcohol stimuli, and although the mean scores are somewhat moderate (around 5), there are also neutral alcohol stimuli that are given high scores by the participants.

This opposite pattern suggests that alcohol and food cues carry different motivational meanings for our low-consumption sample. While food likely evoked a broadly shared appetitive response, alcohol cues may have been perceived less positively, possibly reflecting lower personal relevance or mild avoidance. Higher ratings for some “neutral” alcohol images indicate that these were not entirely affectively neutral for all participants.

### 4.2 Postural displacement indices

With regard to the average postural responses recorded in the various experimental conditions, we observed non-significant modulation for the COP-AP and COP-ML indices. However, the SD and Path-Length indices of COP displacements in these same directions were significantly modulated by incentive (food vs. alcohol) and valence (incentive or neutral) factors. A comparable set of results was recently obtained in a wholly distinct functional context, namely the perception of pleasant environmental scenes (Akounach et al., 2025).

#### 4.2.1 In response to food stimuli

We report a significant increase of SD-COP-AP, SD-COP-ML, AP-Path and ML-Path lengths as compared to neutral-food stimuli. These results show the pertinence of the SD-COP and Path-Length displacement to capture biomarkers of appetizing food stimuli (in opposition to COP displacement *per se*) processing. These can be interpreted as an increase in postural instability in response to the presentation of appetitive food stimuli. In the context of the perception of pleasant environmental scenes, recent observations revealed analogous trends in the active viewing condition, wherein participants were instructed to envision themselves as actors in the scene being presented. Conversely, during passive viewing of these scenes, a decrease in these same cues was recorded (Akounach et al., 2025). As a reminder, in the only study available in the scientific literature, Brunyé et al. (2013) reported a constant and progressive tendency to approach images of appetizing foods.

In line with previous studies using aversive (Azevedo et al., 2005; Facchinetti et al., 2006), empathy-for-pain (Lelard et al., 2013), sexual motivation (Mouras et al., 2015), and postural-threat paradigms (Lelard et al., 2019), we observed changes in postural cues consistent with automatic motor-affective coupling. For food, the observed postural instability may be compatible with the early, implicit components of an embodiment process linking emotion and motor output (Akounach et al., 2025). Prior work supports the automatic nature of this link in response to appetitive food cues, as reflected in peripheral measures such as attentional bias, positive evaluation, craving intensity, and motor readiness (Brignell et al., 2009; Brockmeyer et al., 2015; di Pellegrino et al., 2011; Seibt et al., 2007; Veenstra and de Jong, 2010), as well as in neural correlates (Bertonatti et al., 2021; Roefs et al., 2018; Simmons et al., 2005). While our findings are compatible with this interpretation, further work combining posturography with complementary methods is needed to directly confirm the proposed embodiment mechanisms.

#### 4.2.2 In response to alcohol stimuli

Alcohol images elicited a significant decrease in SD-COP-ML and ML-Path length (with similar but non-significant trends in the AP plane) compared to neutral-alcohol stimuli. These results support the value of SD-COP and Path-Length indices, rather than COP displacement per se, for capturing postural modulation by alcohol-related cues. To our knowledge, only one prior posturography study on alcohol cues found no significant postural differences between alcoholic and sexual stimuli, either in patients with alcohol-use disorders or in healthy controls (Noël et al., 2021). In our healthy sample, alcohol cues appeared to induce a pattern more consistent with a rigidification-type response, possibly reflecting ambivalence between approach and avoidance tendencies. This interpretation remains tentative and suggests that embodiment processes for alcohol may differ in nature or efficiency from those observed for food cues.

It is not appropriate to review the extensive theoretical and practical debates on psychological mechanisms (e.g., approach, inhibition) and their articulation (i.e., parallel or interdependent) that have been conducted in research on alcohol and addiction in this context. However, we find it interesting to note that for alcohol per se, posturography can capture biomarkers of alcohol appetite in the healthy subject, which in this case seems to resonate with ambivalence (between approach and avoidance) and demonstrate a different embodiment that is inoperative or, at the very least, different from that operating for food.

#### 4.2.3 Synthetical interpretation

Our mean results argue for the relevance of posturography to discriminate behavioral responses to appetitive food and alcoholic stimuli respectively, but also to provide biomarkers of the difference in processing in the same subjects between these two types of incentives. This type of approach may, we believe, be of clinical interest for monitoring the evolution over the course of treatment of these two types of marker. Mainly, this study seems to us to show clearly, along with what has been shown in other functional contexts, that embodiment is a central process for the appreciation of appetitive stimuli and that it does not function or functions differently in the processing of stimuli that may be judged more ambivalently, such as alcohol. Whereas for food, our data resonate with an implicit, automatic embodiment mechanism for food stimuli, it would be particularly interesting for alcohol to identify the type of cognitive lever (along the lines of perceptual or cognitive immersion, the importance of which appears in other functional contexts) that would modulate the postural responses to alcohol (increase or decrease of the appearing freezing response).

### 4.3 Temporal dynamics

Several studies have shown the importance to take into account the dynamic of the postural response to apprehend its whole complexity in different functional contexts (empathy for pain, Mouras and Lelard, 2018; pleasant landscapes processing, Akounach et al., 2025). This is particularly true for the postural index COP-AP that is the most cited to index approach-avoidance type behaviors. The postural responses exhibited under various experimental conditions are informative due to the dynamics observed. In the functional context of processing pleasant environmental scenes (Akounach et al., 2025), an effect was demonstrated at the seventh second of stimulus presentation, suggesting a rather late effect. By taking into account the overall dynamics of the postural response under the various experimental conditions, we can provide additional elements of understanding. The incentive conditions (both food and alcohol) are above (in terms of COP displacement in the forward direction) the neutral conditions (Food>Alcohol>Neutral-food>Neutral-alcohol). Looking at the whole dynamic, we report an inversion in the late component of the postural response with neutral conditions passing over incentive ones. If we can make an analogy with the processing of pleasant landscapes recently studied, things are similar for food and pleasant landscapes but the temporality looks like different: for pleasant landscapes, data pledge for a long process of embodiment whereas for food appetitive stimuli, the embodiment process should be much more rapid, automatic and maybe instinctive.

While our postural results can be interpreted within an approach–avoidance framework, they should be considered as implicit indices of motivational tendencies rather than direct measures of approach or avoidance behavior. This interpretation is in line with previous work showing that subtle postural adjustments in response to emotional stimuli can reflect underlying affective and motivational processes, but does not in itself constitute direct behavioral evidence. However, it should be noted that a significant number of studies in the literature have considered posturography to be a valid biomarker of approach-avoidance behavior developed in particular towards motivational targets (Mouras et al., 2023).

Several limitations should be acknowledged. First, while alcohol-related stimuli are inherently multidimensional, potentially evoking affective, cognitive, and socially embedded associations, the present study used decontextualized, standardized images for both alcohol and food in order to compare them under strictly equivalent presentation conditions. This methodological choice allowed us to treat them as two categories of motivational targets and to isolate their core embodied salience. However, it also limits ecological validity, and future studies should incorporate measures of social context, stress sensitivity, or addiction severity to better capture the complexity of alcohol-related cue reactivity.

Second, although participant exclusion criteria and outlier handling procedures are now explicitly detailed in the revised Methods, some degree of heterogeneity in alcohol use remained within the final sample. Our initial approach was to conduct global analyses, while measuring individual alcohol consumption with the AUDIT to include as a covariate if necessary. It is important to note that overall AUDIT scores were relatively low compared to the full possible range, suggesting that motivational differences between participants were likely limited. Given our relatively large sample size for a posturography study, we subsequently conducted exploratory subgroup analyses by splitting the sample at the median AUDIT score, which are reported in the Supplementary materials. These analyses provide preliminary indications of potential differences between lower- and higher-AUDIT participants, but are necessarily constrained by statistical power. Future studies should recruit more homogeneous or deliberately contrasted subgroups to better isolate the influence of alcohol use severity on embodied responses to alcohol cues.

Finally, while EEG data were recorded simultaneously as part of a broader experimental protocol, no EEG analyses are reported here. These will be presented in a separate publication focusing on the temporal neural dynamics of motivational processing. This choice ensures that the present article remains focused on postural and affective measures while maintaining transparency regarding the scope of the reported results.

## Data Availability

The raw data supporting the conclusions of this article will be made available by the authors, without undue reservation.
