# Supplementary material for: Postural correlates of visual incentives: application to food and alcohol stimuli
Source: Front Psychol. 2025 Nov 3;16:1612425. doi: 10.3389/fpsyg.2025.1612425 (PMC12620430; doi:10.3389/fpsyg.2025.1612425)
Supplement: Supplementary file 1 [file Data_Sheet_1.pdf]

Supplementary Online Material

15 avril 2025

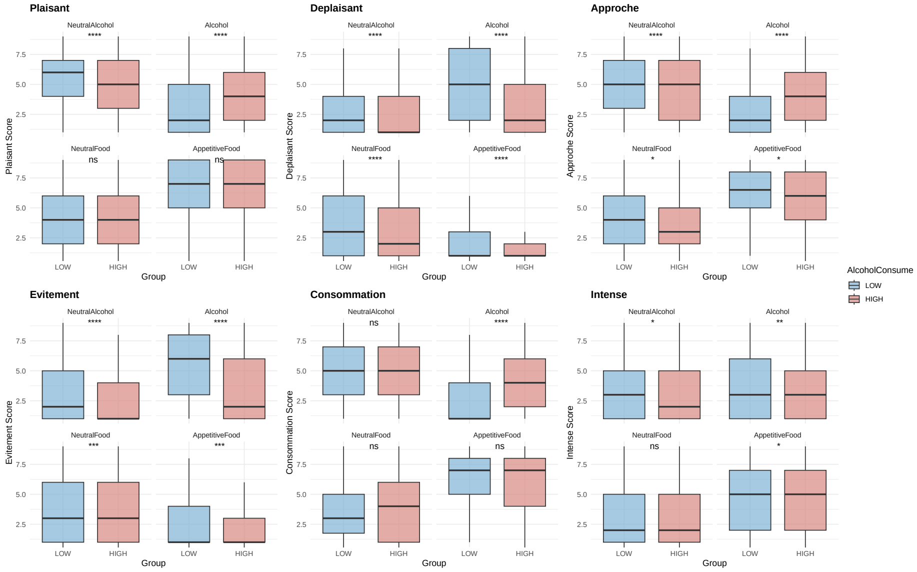

FIGURE 1 – Subjective ratings for alcohol- and food-related stimuli by alcohol consumption level (ns=not significant, * $p < 0.05$, ** $p < 0.01$, *** $p < 0.001$, **** $p < 0.0001$).

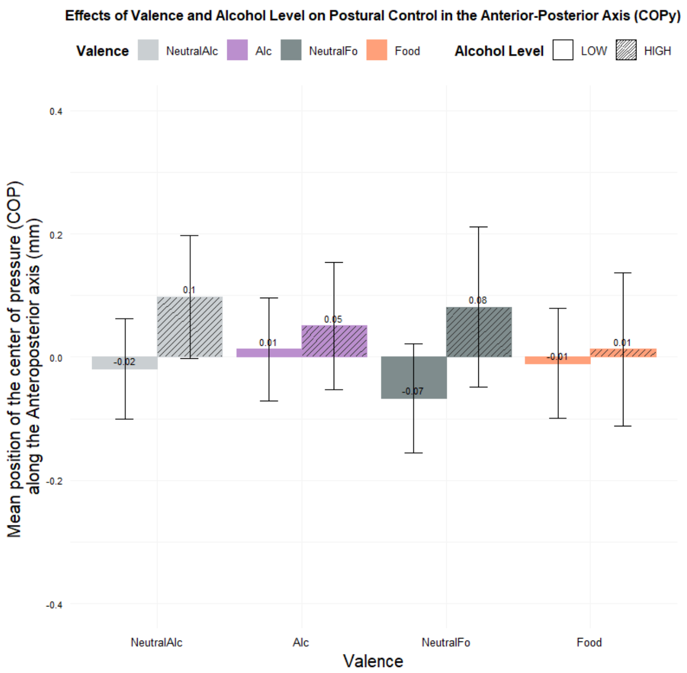

FIGURE 2 – Mean anterior-posterior displacement (COPy) across valence categories and alcohol consumption levels.

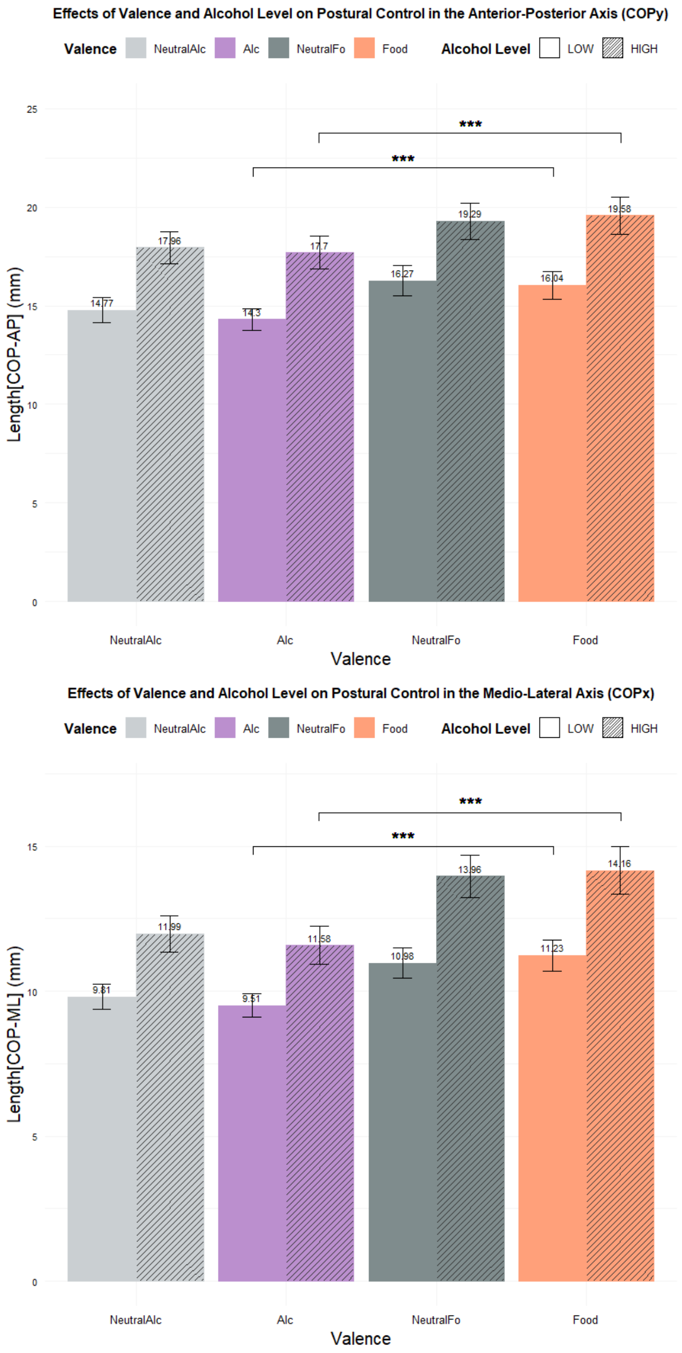

FIGURE 3 – Postural sway (COP length) in anterior-posterior (a) and medio-lateral (b) axes by stimulus valence and alcohol level *** $p < 0.001$.

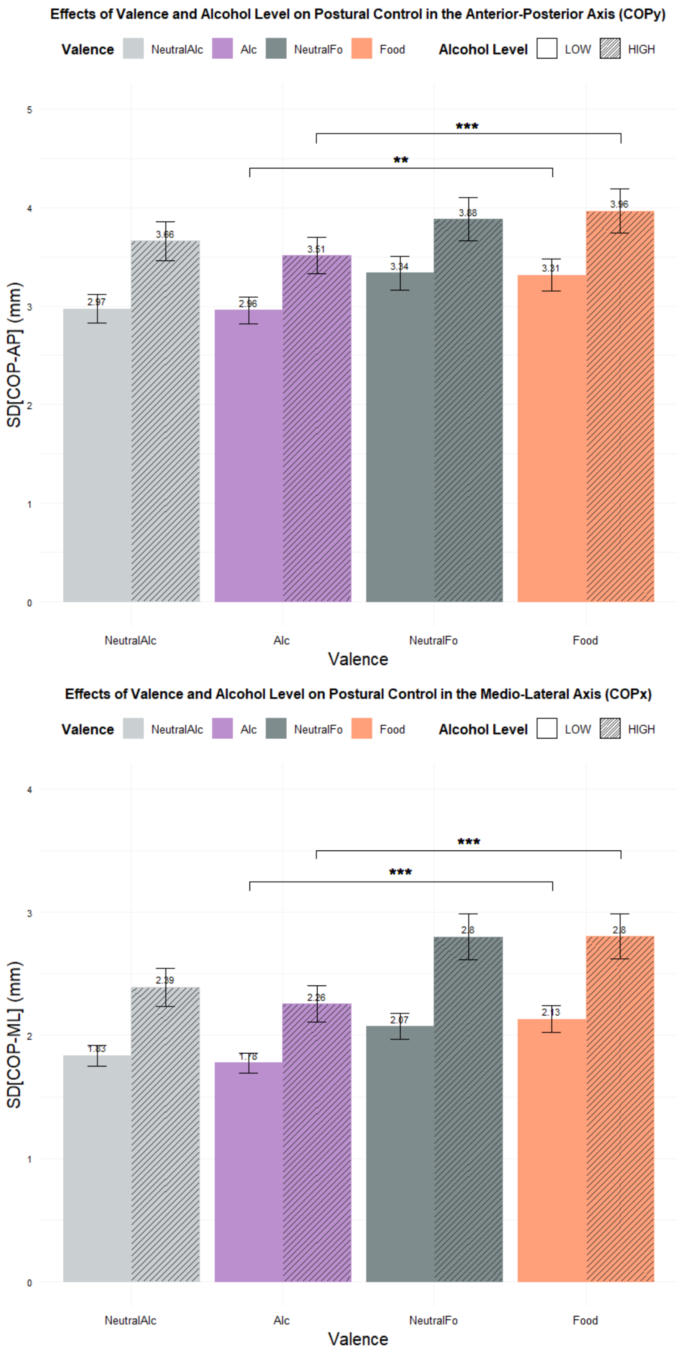

FIGURE 4 – Postural sway variability (SD of COP) in anterior-posterior (a) and medio-lateral (b) axes across valence and alcohol level (** $p < 0.01$, *** $p < 0.001$).
