# Supplementary material for: Postural correlates of visual incentives: application to food and alcohol stimuli
Source: Front Psychol. 2025 Nov 3;16:1612425. doi: 10.3389/fpsyg.2025.1612425 (PMC12620430; doi:10.3389/fpsyg.2025.1612425)
Supplement: Supplementary file 2 [file Data_Sheet_2.pdf]

Stimuli databases selection procedure

September 1, 2025

In designing the experimental paradigm, four conditions were considered: Alcohol, Neutral Alcohol, Appetitive Food, and Neutral Food. Several critical factors were taken into account when selecting the relevant stimuli from existing databases.

Firstly, a uniform white background was used to ensure that participants focused solely on the stimuli while minimizing potential distractions from background elements. Secondly, particular attention was given to selecting realistic and naturally appearing images. Artificial-looking images, such as objects without shadows or those that did not rest on a surface, were avoided, as they might introduce an unnatural visual perception. Thirdly, to prevent pixelation, image databases containing high-resolution visuals were preferred, considering that some databases included low-resolution images. Additionally, images with excessively bright white backgrounds were converted to grayscale using MATLAB to maintain visual consistency.

Another crucial aspect of stimulus selection was the definition of neutral food and neutral alcohol stimuli. Neutral food was defined as plain food items without any specific color, taste, or presentation emphasis. Examples include fruits and vegetables without garnishes or sauces (e.g., broccoli, zucchini, boiled vegetables without spices or condiments), plain grains (e.g., rice, oats, quinoa), unsweetened oatmeal or porridge, simple protein sources such as boiled eggs, unsweetened bread and whole grains, clear soups without distinctive ingredients or sauces, tofu, plain pasta, rice, or boiled potatoes, and unsweetened yogurt without fruit or sweeteners (Toet, Kaneko, Ushiama, et al., 2018). On the other hand, neutral alcohol stimuli were defined as non-alcoholic beverages.

To ensure the appropriate selection of alcohol, neutral alcohol, appetitive food, and neutral food stimuli, three well-established databases were utilized: CROCUFID (Cross-Cultural Food Image Database; Toet, Kaneko, De Kruijf, et al., 2019) , ABPS

(Amsterdam Beverage Picture Set; Pronk et al., 2015), and the Australian Beverage Picture Set (Onie et al., 2020).

CROCUFID (Cross-Cultural Food Image Database) is a comprehensive dataset that includes images of foods from different cultures. It serves as a valuable resource for researchers studying food preferences, eating habits, and cultural differences. This database used a standard photography protocol. As a result, high-resolution images (1037x691 pixels) were obtained with all food items viewed on a standard background (a white plate and a fixed background) from a fixed viewing angle (45 degrees). They added images of foods with different appetite levels (fresh, foreign, moldy or rotten, spoiled and partially consumed) to create sufficient variability in valence and arousal. CROCUFID was used to select neutral food and appetite-provoking food stimuli due to the fixed background, realistic stimuli, the presentation of foods on a plate, the presence of shadows on the food, the variety of foods and the high quality of the images.

The Amsterdam Beverage Picture Set (ABPS) is a database focused on visualizing beverages, commonly used in studies exploring alcohol consumption, thirst perception, and the psychological effects of beverage preferences. This database contains 44 alcoholic and 33 non-alcoholic beverage images. The database includes beer, wine, spirits and alcopops as alcoholic beverage stimuli. Non-alcoholic beverages include water, soft drinks and miscellaneous. Object angles and lighting were controlled when taking photographs. The dimensions of the images are 500 x 500 pixels. The high-resolution images allow for a clear analysis of packaging designs and liquid contents. ABPS also features different presentation formats, such as beverages served in glassware, bottles, or cans, making it a valuable resource for studies on consumer perception.

Due to insufficient visuals in the ABPS database, additional databases were consulted and the Australian Beverage Picture Set was preferred. It includes a diverse range of drinks, such as water, fruit juices, soft drinks, and alcoholic beverages. The images are carefully captured with uniform lighting and a standardized background to minimize visual variability in research settings. These high-resolution images showcase beverages in both packaged and open forms, making the data-set a useful tool for studies examining consumer behavior, the health impacts of beverage choices, and the perception of different drinks.

References

Onie, Sandersan et al. (2020). "Validation of the Australian beverage picture set: A controlled picture set for cognitive bias measurement and modification paradigms." In: *Australian Journal of Psychology* 72.2, pp. 223–232.

Pronk, Thomas et al. (2015). "Validation of the Amsterdam Beverage Picture Set: A controlled picture set for cognitive bias measurement and modification paradigms." In: *Alcoholism: Clinical and Experimental Research* 39.10, pp. 2047–2055.

Toet, Alexander, Daisuke Kaneko, Inge De Kruijf, et al. (2019). "CROCUFID: A cross-cultural food image database for research on food elicited affective responses." In: *Frontiers in psychology* 10, p. 58.

Toet, Alexander, Daisuke Kaneko, Shota Ushiama, et al. (2018). "EmojiGrid: A 2D pictorial scale for the assessment of food elicited emotions." In: *Frontiers in psychology* 9, p. 2396.
