# Supplementary material for: Postural correlates of visual incentives: application to food and alcohol stimuli
Source: Front Psychol. 2025 Nov 3;16:1612425. doi: 10.3389/fpsyg.2025.1612425 (PMC12620430; doi:10.3389/fpsyg.2025.1612425)
Supplement: Supplementary file 3 [file Data_Sheet_3.pdf]

Supplementary S2 — A priori power and sensitivity analyses

28 août 2025

Model : Repeated-measures ANOVA (within-subject factor, 4 levels)

Inputs : $\alpha = .05$; $1–\beta = .80$; effect size $f = 0.20$; $\varepsilon = .75$

Assumptions : moderate correlation among repeated measures ; conservative ε to account for potential sphericity violations

Output : Required $N \approx 52$

Observed : N = 55 (adequate)

Sensitivity (paired comparisons)

Model : Paired t-tests (two-tailed) for planned within-subject contrasts

Inputs : N = 55 ; $\alpha = .05$; $1–\beta = .80$

Output : Detectable within-subject effect $dz \approx 0.38–0.40$

Note on effect-size choice : We selected f = 0.20 as a conservative small-to-medium effect size for posturographic modulation by motivational cues, to avoid over-optimistic power estimation.
